# Effect of cement volume on biomechanical response of a spine segment treated with a PEEK polymer implant: a finite element comparative study with vertebroplasty

**DOI:** 10.3389/fbioe.2024.1399851

**Published:** 2024-06-11

**Authors:** Cécile Vienney, Ridha Hambli, Reade De Leacy, François H. Cornelis

**Affiliations:** ^1^ Hyprevention, Research and Development, Pessac, France; ^2^ University of Orléans, University of Tours, INSA CVL, LaMé, Orléans, France; ^3^ Neurosurgery, Icahn School of Medicine at Mount Sinai, New York, NY, United States; ^4^ Memorial Sloan Kettering Cancer Center and Weill Cornell Medical College, Radiology Department of Radiology, New York, NY, United States

**Keywords:** lumbar spine, vertebral compression fractures, implant, vertebroplasty, cement, dose, finite element

## Abstract

In the current study, a 3D finite element study was performed to investigate the biomechanical response of an osteoporotic spine segment treated with a novel transpedicular implant (V-STRUT^©^, Hyprevention, France) made of PEEK (polyetheretherketone) material combined with either injections of 2, 3, 4, 5 and 6 cc of cement. The objective was to assess numerically the biomechanical performance of the implant in combination with different doses of the injected bone cement and to compare its performance with the gold standard vertebroplasty (VP) technique. A female (69 yo) was selected and a 3D finite element model of an osteoporotic spine segment was built based on a Computed Tomography (CT) scan performed from T12 to L2 with corresponding intervertebral discs and ligaments. A heterogeneous distribution of bone material properties was assigned to the bone using grey scale levels. Bilateral ellipsoid geometries of the inserted cement were retained for the V-STRUT and VP models based on experimental observation performed on different patients treated with the V-STRUT device. The current study demonstrated an optimal dose of 4 cc of bilaterally injected cement for the V-STRUT and VP techniques to restore the treated segment and confirmed that the V-STRUT device in combination with bone cement is superior to VP alone in establishing the normal stiffness and in reducing the applied stress to the immediately adjacent vertebral levels.

## 1 Introduction

With aging, bone quality decreases due to osteoporosis, increasing the risk of vertebral compression fractures (VCFs) (Melton, 1997; Bow et al., 2012; Balasubramanian et al., 2019). VCFs are common in osteoporotic individuals and are more often the result of daily activities rather than high force trauma. The prevalence of VCFs increase with age ranging between 20% and about 65% in older woman. The gold standard interventional technique (VCF treatment) is vertebroplasty (VP). VP involves the injection of an amount of bone cement into the fractured vertebral body with the goal of restoring the strength and the stiffness of the fractured vertebra (Zhang et al., 2015, 2017). However, this technique is often associated with leakage of cement at the target vertebra into the adjacent intervertebral disc/s or soft tissues (Papanastassiou et al., 2014).

**FIGURE 1:**
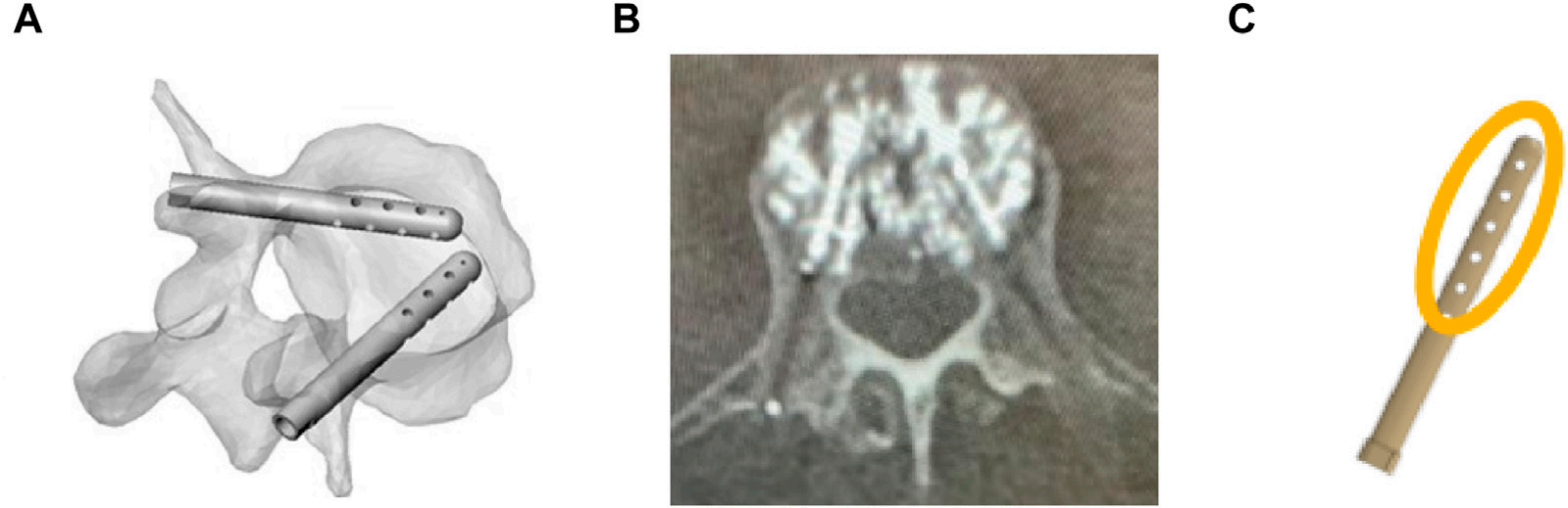
**(A)** Representation of V-STRUT before cement injection in vertebral body. **(B)** V-STRUT with bone cement, **(C)** Schematic representation (orange) of the cement distribution around the V-STRUT implant.

To improve VCF treatment, a new implantable technique and device (V-STRUT^©^) have been developed in order to share load between the anterior and posterior column with the aim of reducing stress in the posterior column, to restore vertebral strength, stabilize the fractured vertebra and prevent the progression of postoperative fractures (Figure 1a). Two implants, made of PEEK polymer (PEEK Optima^®^, INVIBIO), are inserted in the vertebral body through the pedicles and combined with the injection of bone cement (F20^®^, Teknimed, S.A.S, France) (Figure 1b). When the bone cement hardens, the implants are fixed in the vertebra and then these will stay *in situ* (Figure 1b). The implants are cannulated to allow a uniform cement dispersion (Figure 1c).

Several numerical studies were developed in the past in order to investigate the role of the cement after vertebral augmentation (Belkoff et al., 2001; Liebschner et al., 2001; Jasper et al., 2002; Morgan et al., 2003; Kurtz et al., 2005; Trout et al., 2006; Rohlmann et al., 2010; Elmasry et al., 2018; Peng et al., 2018). Typically, different morphologies, volumes and mechanical properties of cement were proposed by different authors (Liebschner et al., 2001; Song et al., 2022). Bone cement was placed into the augmented vertebral bone model using CAD functions to assemble the cement with the vertebral body with bilateral and unilateral injection patterns. Elmasry et al. (2018) developed an enhanced 3D FE study to evaluate and compare the immediate post-operative biomechanical performance of stand-alone percutaneous pedicle screw fixation (PPSF), stand-alone Balloon kyphoplasty, and KP-augmented PPSF procedures. The authors showed that no significant performance was obtained between stand-alone PPSF and KP-augmented PPSF procedures. Zhang et al. (2022) investigated the stress changes between different bone cement forms and injection volumes in adjacent vertebrae after percutaneous kyphoplasty using 3D FE analysis of osteoporotic lumbar vertebral body. The authors extracted different morphological bone cement models from CT scans of three female patients and combined these cement geometries with 3D FE vertebral body models. Gao et al., 2022 reconstructed 3 D models of bone cement based on CT scans of treated patients with.

The optimal amount of bone cement to inject into a fractured vertebra has not been clearly established for various restoring techniques. Most studies have recommended a range of 4–8 mL of injected cement (Belkoff et al., 2001; Nieuwenhuijse et al., 2012).

The present study investigated the role of the implant and impact of cement volume on the biomechanical response of the augmented vertebra and the spine segment. Therefore, the objective was twofold: i) Assess numerically the efficacy of the V-STRUT^©^ transpedicular implant in relation with different bone cement doses and ii) Compare the performance of the device with the gold standard VP technique.

## 2 Methods

**FIGURE 2:**
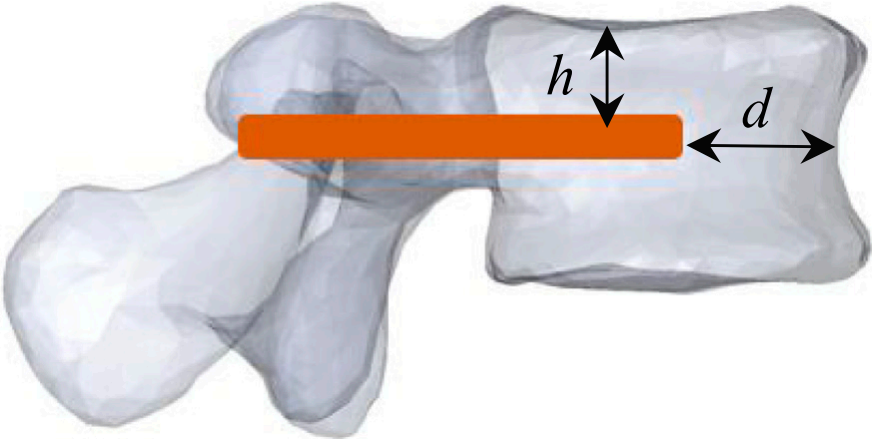
Model V-STRUT: optimal position of the V-STRUT implant. *d* = 5 mm: distance from anterior wall, *h* = 15 mm: height from superior endplate.

In a previous work (Hambli et al., 2024), we demonstrated that the optimal position of the V-STRUT implants within the treated vertebra allowing a uniform stress distribution in the augmented spine segment and the treated vertebral body correspond to distance from anterior wall (*d* = 5 mm), and height from superior endplate (*h* = 15 mm) (Figure 2). In the current work, three finite element models were developed to investigate the biomechanical response of an osteoporotic spine segment treated with the V-STRUT and VP techniques combined with the bilateral injection of a total five volumes of cement (2, 3, 4, 5 and 6 cc) for each model and a non-treated osteoporotic model (reference model).

Model V-STRUT: Spine segment with inserted device (*h* = 15 mm, *d* = 5 mm) (Figure 2), Model VP: Spine segment without implant and with cement (vertebroplasty), Model Ref: Spine segment alone without implant and without cement.

The FE model (Model Ref), without implant and without cement was retained as the benchmark model to show the differences after the V-STRUT and VP treatments. For each (V-STRUT and VP) models, five different cement doses (2, 3, 4, 5 and 6 cc) were investigated resulting in a total of 10 different cases. In total with including the Model Ref, 11 simulations were then performed and stiffness and stress distribution within the spine segment were computed for analysis and comparison.

### 2.1 Spine segment finite element model

**FIGURE 3:**
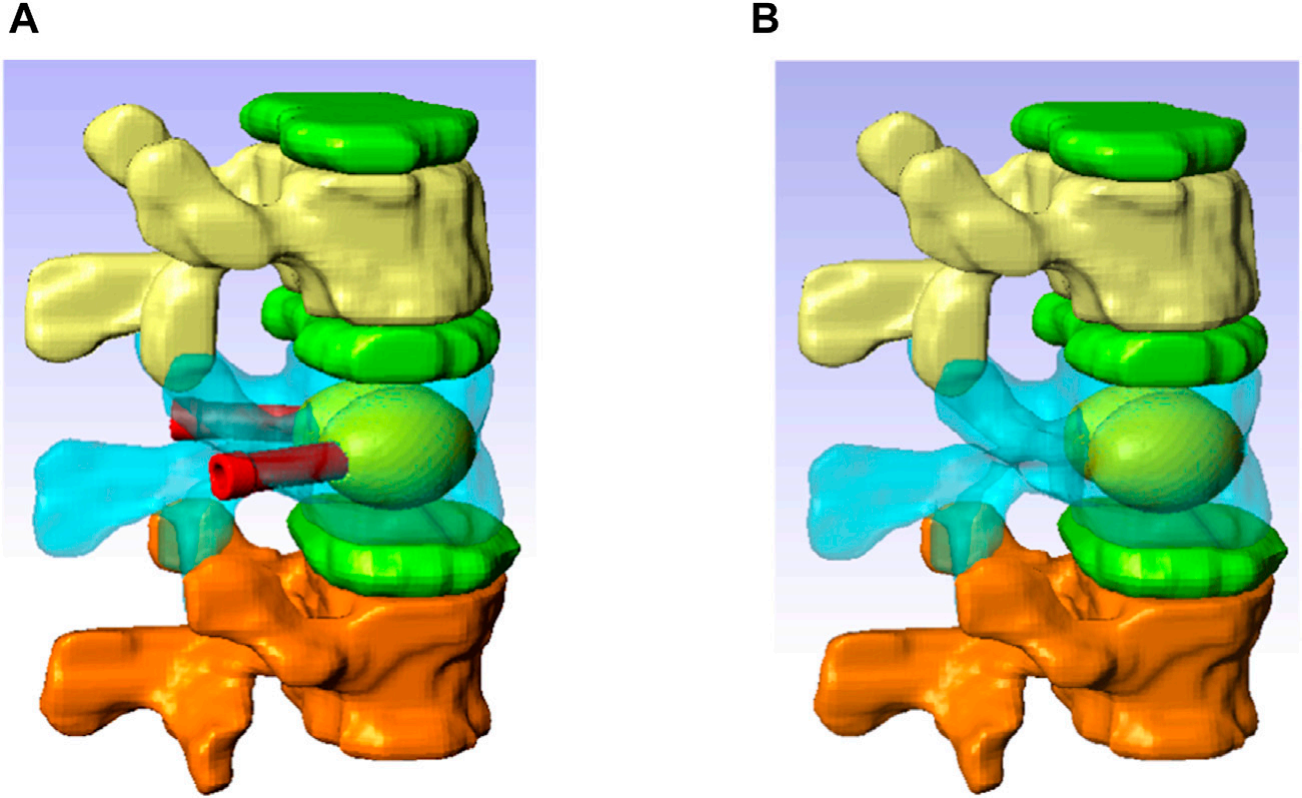
Spine segment models with inserted the V-STRUT device and the cement **(A)** and vertebroplasty **(B)**.

The 3D FE model was built based on a computed tomography (CT) scan of the thoraco-lumbar spine of an osteoporotic patient (Female, 69 yo). The geometry was generated using the software ScanIP (Simpleware, Exeter, United Kingdom) (Figure 3).

**FIGURE 4:**
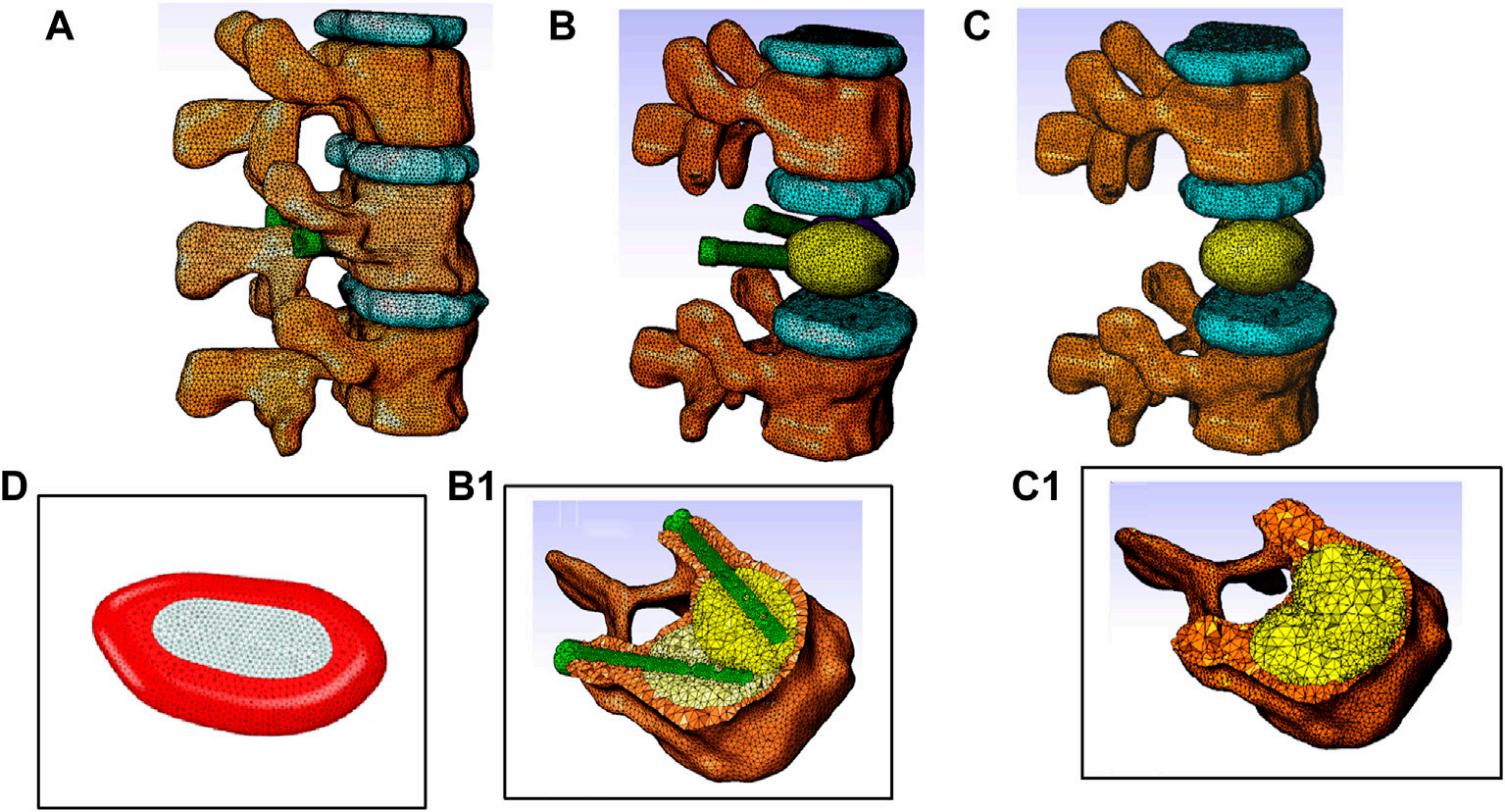
Finite element models of the thoraco-lumbar spine segment composed of T12, L1 and L2 vertebrae, intervertebral discs and ligaments. **(A)** General view, **(B)** Model V-STRUT (augmented vertebra is hidden for clarity) with 6 cc cement. **(B1)** Details of the implants and cement within the vertebra. **(C)** Model VP (augmented vertebra is hidden). **(C1)** Details of the bilateral 6 cc cement within the vertebra. **(D)** Intervertebral disc is composed by two regions: Annulus fibrosus material (red) and nucleus pulposus material (light grey).

The model consists of a spine segment (SS) composed of three consecutive vertebrae (T12, L1 and L2), three intervertebral discs and spinal ligaments (Figure 3b). Two CAD implants and cement geometries were imported and inserted in a second step in the middle vertebral body through the pedicles to generate two models representing two clinical techniques: V-STRUT (Figure 3a) and vertebroplasty (VP) (Figure 3b). The intervertebral discs were modeled using two regions describing the annulus and the nucleus and linear beam elements with specific stiffness representing each ligament (Figure 4d).

### 2.2 Cement finite element model

The high viscosity at which the bone cement is injected limits the uniform diffusion of the bone cement in the whole vertebral body, thus limiting the distribution of the cement into the vertebra and creating an agglomerate of cement localized around the implants with irregular shape depending among other on the spongy bone pattern.

Typical bilateral ellipsoid geometries of the inserted cement were observed based on X-rays of different patients treated with the V-STRUT device (Figure 1b). In the current work, cement ellipsoid capsules were then retained with five different varying volumes representing five injected total cement doses (2, 3, 4, 5 and 6 cc) for bilateral injection pattern (Figure 3a). Bone cement was placed into the L1 spine bone model. The principal axis of each cement volume was designed to be aligned with each implant representing realistic bone cement morphology distributed around the implants. The cement was imported into Simpleware software in CAD format and then assembled with the implants using the assembly commands (Figure 3a). A complete FE model of the cement-reinforced spine segment was obtained by removing excess bone with the Boolean function. An input file was then generated and imported into the Abaqus software to perform the simulations.

Each vertebra was composed of about 110,000 tetrahedral finite elements (about 450,500 elements for the whole model) to represent the smooth surface of the bone (Groenen et al., 2018; Hambli et al., 2023). Frictional contact with relative sliding motion between two contacting surfaces with a friction coefficient of 0.01 was defined between each vertebra/disc surface (Wan et al., 2022). The facet joints were modelled as a frictionless contact with an initial gap of 0.5 mm (Hambli et al., 2024).

Contacts between implants, cement and bone were modelled as fully bonded interfaces without separation during the simulations with a step change from the bone cement and implants domains via shared node bonding of the domains.

### 2.3 Boundary conditions

Cannada and Hill (2014) reported that daily normal load can cause osteoporotic bone fractures. The investigational implant was designed to provide more uniform strength to an osteoporotic vertebra and to prevent the progression of VCFs post-operatively. Therefore, in the current study, the load transfer was limited to the compressive physiological motions of the spine. In the current work, a compressive pressure of magnitude of 1 MPa was applied on the superior endplate of the T12 vertebra Corresponding to the case of jogging (Wilke et al., 1999).

**FIGURE 5:**
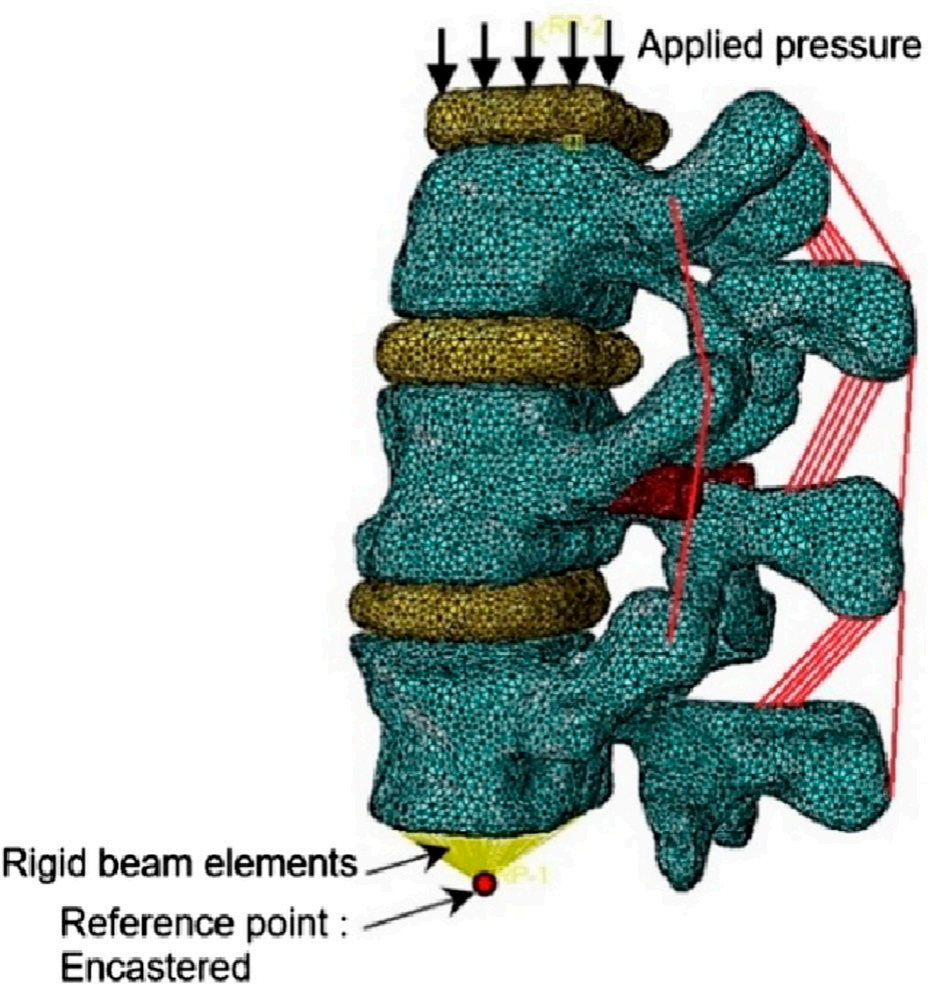
Loading and boundary conditions applied for the simulations.

Reference node were placed at the center of the vertebral body at the inferior vertebra (L2) and this reference point was encastered for the simulations (Figure 5a). The nodes belonging to the endplate surface of the lower vertebral body (L2) were then tied to this reference node with rigid body elements.

### 2.4 Material properties

#### 2.4.1 Bone

**FIGURE 6:**
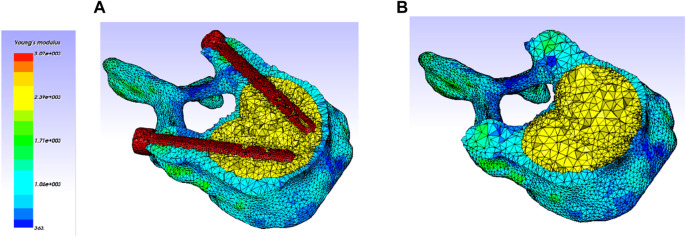
Heterogeneous elastic modulus distribution within the vertebra (Only treated one is kept, adjacent ones were hidden for clarity). **(A)** V-STRUT model (with cement and implants). **(B)** VP model (with cement).

Bone material was modeled with an elastic linear behavior with a heterogeneous distribution of bone material properties assigned to the vertebrae obtained from the grey scale levels. The heterogeneous elastic modulus of the bone material was assigned to each FE of the mesh based on the relationship (Eq. 1) provided by Morgan et al. (2003) as follows (Figure 6):

$$E=15010\rho^{2.18}\;\left(\rho \leq 0.280\right)$$

$$E=6850\rho^{1.498}\;\left(\rho \;>0.280\right)$$ (1)

where ρ (g/cm^3^) denotes the bone density.

#### 2.4.2 Discs

In the current study, the discs were partitioned nucleus and annulus regions (Dreischarf et al., 2014) modeled using the Mooney-Rivlin material model (Wilke et al., 1999; Schmidt et al., 2007).

The properties ( C10 and C01 parameters of the Mooney-Rivlin model) for rather degenerated discs were used (Table 1) in the current study (Schmidt et al., 2007).

**TABLE 1:** Mechanical properties of the materials for different tissues of the spine model.

Tissue	Model	Properties			References
Vertebra	Isotropic linear elastic	$E=15010\rho^{2.18}$		$\left(\rho \leq 0.280\right)$	Morgan et al. (2003)
		$E=6850\rho^{1.498}$		$\left(\rho \;>0.280\right)$	
		ν=0.3			
Discs	Hyperelastic		C01	C10	Schmidt et al. (2007)
		Annulus	0.09	0.12	
		Nucleus	0.045	0.18	
Ligaments	Linear elastic	Ligament		stiffness (N/mm)	Polikeit et al. (2003)
		Longitudinal anterius		210	
		Longitudinal posterius		20.4	
		Supraspinale		23.7	
		Interspinale		11.5	
		Intertransversium		50	
		Flavum		27.2	
		Capsular		33.9	
Cement	Isotropic linear elastic	E=1820 MPa			Robo et al. (2018)
		ν=0.3			
V-STRUT implant	Isotropic linear elastic	E=3600 MPa			Jhong et al. (2022)
		ν=0.3			

#### 2.4.3 Ligaments

The ligaments were represented numerically by beam elements considering their anatomical locations (Figure 5). Each ligament behavior was considered as linear elastic represented by a specific stiffness (Table 1) (Polikeit et al., 2003; Hambli et al., 2023).

#### 2.4.4 Implants and cement

The V-STRUT implants (PEEK material) and the cement injected with the device is of type (F20^®^, Teknimed, S.A.S, France) exhibit both linear isotropic behavior. Young modulus and Poisson ratio of ach material are reported in Table 1.

## 3 Results

The qualitative validation of the proposed 3D FE model was performed in a previous work (Hambli et al., 2023) by comparing the computed compressive stiffness of the present osteoporotic spine segment with published experimental data measured for the L12-T2 osteoporotic spine segment of a female under compressive load (Groenen et al. (2018) (Table 2). The compressive stiffness K is expressed by $K=\frac{F}{u}$ , where u is the axial compressive displacement of the reference point (Figure 5) and F the compressive strength applied to the spine segment.

**TABLE 2:** FE predicted values of stiffnesses for the different spine segment models.

Model	Stiffness (N/mm)
Experiment Groenen et al. (2018)	2,206
Prediction: FE (without implants)	2,736
Prediction: FE (with implants and 2 cc of cement)	3,455

In their investigation, the authors were destructively tested in axial compression twelve two functional spinal units (T6-T8, T9-T11, T12-L2 and L3-L5).

One can notice that predicted results (2,736 N/mm) was in the range of the experimental one (2206 N/mm) reported in the study of Groenen et al. (2018). Despite the current investigated spine segment was different from the study of Groenen et al. (2018). Considering the anatomical complexity of the lumbar spine, such a validation is satisfactory for general conclusions for the purpose of current work.

**FIGURE 7:**
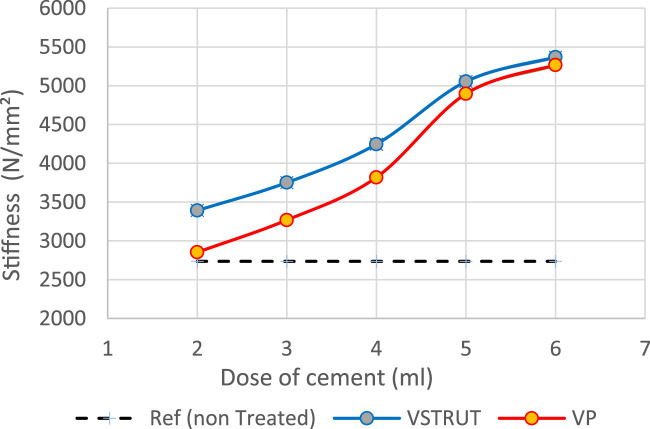
Predicted stiffness variation of the spine segment versus bone cement dose for V-STRUT, VP techniques and non-treated one (Model Ref).

Current predicted results indicated that the stiffness of the spine segment increased with the increase of the cement dose (Figure 7).

Our results showed that VP generated a lower stiffness increase compared to the V-STRUT device. This can be explained by the reinforcement of the vertebra by the stiffness of the assembly composed by the device and the cement that is not the case for the VP solution.

**FIGURE 8:**
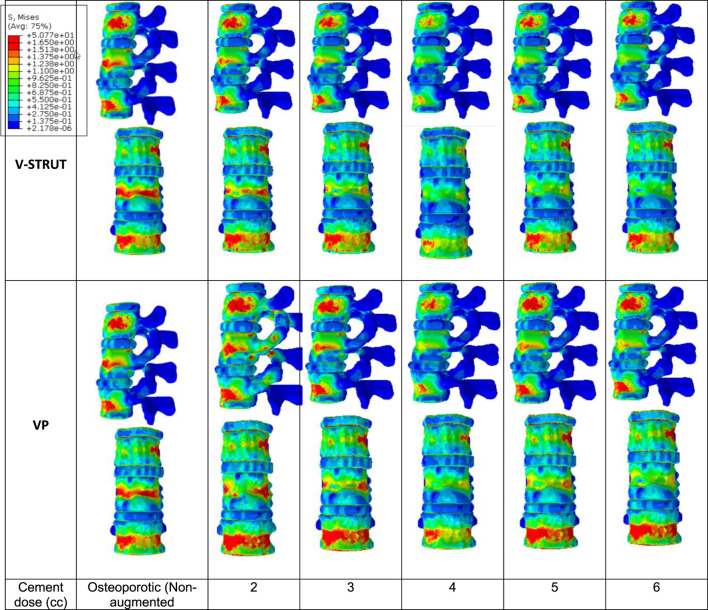
Stress distribution applied to the cortical bone for the osteoporotic non-treated model (Model Ref) and for both V-STRUT and VP models with five different doses of the injected cement.

Figure 8 depicts the distribution of the von Mises stress applied to the cortical bone for both V-STRUT and VP models with five different doses of the injected cement and the osteoporotic non-treated model (Model Ref).

It can be observed that the maximum stress occurs for the non-treated model (Model Ref) concentrated mainly in the cortical bone of the middle vertebra. V-STRUT and VP treatment reduced the stress contour significantly compared to the non-treated one with a lowest stress value corresponding to bone cement of 4 cc dose for both techniques. The dose of the cement plays a main role on the stress distribution on the cortical bone of the augmented vertebra and the adjacent ones.

The results shows that the insertion of the implants-cement assembly into the augmented vertebra reinforced its stiffness (Figure 7) and hence, played a role as a barrier for stress transfer to the adjacent vertebra (Figure 8). Our results indicate that the adjacent vertebra undergoes higher stress level for VP compared to the VSTRUT treatment.

**FIGURE 9:**
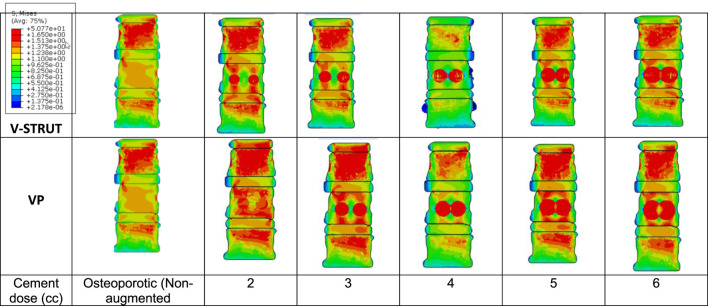
Stress distribution applied to the spongy bone for the osteoporotic non-treated model (Model Ref) and for both V-STRUT and VP models with five different doses of the injected cement.

In Figure 9 the stress contour in the spongy bone (longitudinal cross section) is presented in relation with the cement volume variation.

One can notice that the injected cement volume plays an important role on the stress level and distribution on the spongy bone, the implants and the intervertebral discs of the treated vertebra and adjacent ones. Current results indicate that stress exhibits high value with low values of cement doses (2 and 3 cc). Nevertheless, 4 cc generated a reduced stress for both techniques. With the increase of the cement dose beyond this value, the maximum stress is transferred to the cement and the implants and no significant stress variation can be observed when the dose exceed 4 cc. In contrast, the stress level in the complex implant/spongy bone and at the endplates increases significantly. When comparing V-STRUT and VP, predicted results indicated that V-STRUT approach generated a lower stress value for the treated and adjacent vertebrae.

**FIGURE 10:**
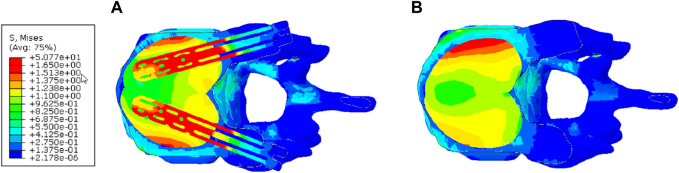
Distribution of the stress in the axial middle section of the augmented vertebra (Case of 4 cc of cement dose). **(A)** V-STRUT. **(B)** Vertebroplasty.

Figure 10 depicts the stress contour in an axial sectional view of the augmented vertebra (case of 4 cc cement dose). The VSTRUT solution transferred uniformly a part of the load to the posterior column indicating that the compressive load is shared with interior and exterior column which is not the case with the VP solution.

**FIGURE 11:**
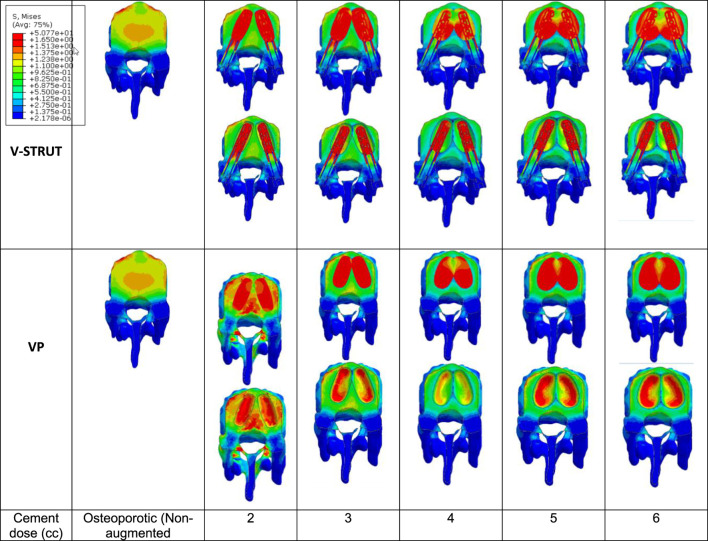
Stress distribution at the interface bone/cement-implant assembly (cross section of the treated vertebra).

When comparing the VP and V-STRUT techniques (Figure 11), our results indicated that both approaches generated different contact stress distribution patterns at the cement/bone interfaces when the cement dose varies. Nevertheless, the transferred stress applied to the spongy bone for augmented vertebra is higher for VP when compared to the V-STRUT in the region of bone/cement contact region.

For the V-STRUT technique, the simulations indicated that a part of the stress is transferred to the implants (Figure 9) that undergoes bending deformation (Hambli et al., 2023). This can be viewed as a cushioning factor. In combination with the cement, the implants contribute to the vertebral strengthening and stabilization and to the cushioning of the applied compressive load.

**FIGURE 12:**
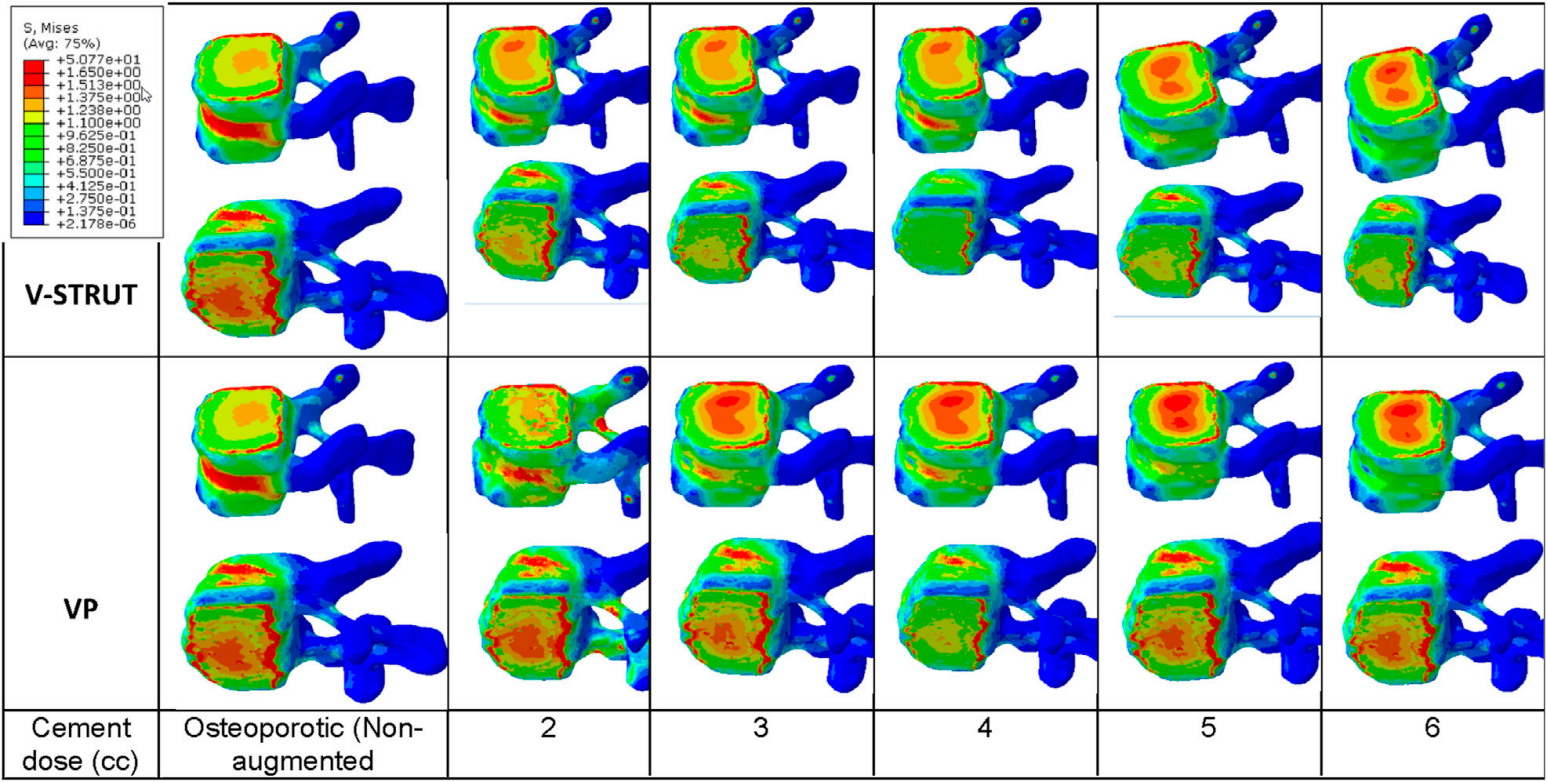
Stress contour on the endplates of the treated vertebra. The non-augmented vertebrae were hidden for a better visualization.

With the increase of the injected dose of the bone cement, the change in stress distribution and magnitude were apparent in the endplates of the augmented body (Figure 12). Note that the non-augmented vertebrae were hidden for a better visualization.

One can notice that 4 cc cement volume ensured reduced stress on the upper and lower endplates for the V-STRUT device. For VP solution, 2 cc of cement generated a lower stress value at the upper endplates and 4 cc of cement for the lower endplates. The whole simulations show that V-STRUT technique exhibits the lowest stress increase compared to the VP one.

## 4 Discussion

The biomechanical effects on osteoporotic spine segments treated with the novel PEEK transpedicular implant (V-STRUT^©^) combined with the bilateral injection of PMMA cement was not yet fully investigated in regard with the cement dose.

In the current investigation, 3D FE models were developed of a lumbar spine segment treated with V-STRUT device combined with the bilateral injection of five different cement doses (2, 3, 4, 5, 6 cc) representing clinical possibly injected amount of cement in the implants. The objective was to assess numerically the biomechanical performance of the implant and to compare its efficacity with the gold standard vertebroplasty (VP) technique.

Predicted results indicated that both V-STRUT and VP techniques increased the stiffness of the spine segment with the injection of the cement when compared to the non-treated spine. Figure 7 showed that the stiffness increased non-linearly with the cement dose with predicted higher-level values when V-STRUT is used. A stiffness gradient of about 1000 N/mm^2^ can be observed in the range of cement dose of 4-5 cc. When the cement dose is greater than about 4-5 cc for both techniques, the variation is limited indicating that at high values, the cement dose plays a limited role. This can be explained by the fact that filling the treated vertebra with high dose of cement generates almost homogeneous assembly and may results in a saturation phenomenon which limit the stiffness variation and may increase the risk of cement leakage. Several studies reported that excessive cement filling of treated vertebra does not produce optimal biomechanical effects, and a reasonable treatment strategy for the vertebral body includes the use of a small, symmetrically distributed amount of bone cement filling (Liebschner et al., 2001; Tseng et al., 2008; Xie et al., 2015). The FE study of Liebschner et al. (2001) found that only a small volume of injected bone cement (about 3.5 cc) is necessary to restore compressive stiffness of the damaged vertebral body to its value before damage and that increase in the injected volume beyond a value of about 4.75 cc generated limited increase of the stiffness.

Our results showed that the VP generated a lower stiffness increase compared to the vertebra augmented with the V-STRUT device. This can be explained by the reinforcement of the vertebra by the stiffness of the assembly composed by the device made of PEEK and the cement.

We found that a total of 4 cc (correspond to 15% of the vertebral body) of injected bone cement for both V-STRUT and VP is sufficient to restore the injured spine segment. This result is in conformity with the studies of Brojan et al. (2015) which found based on biomechanical study that the stiffness of the treated vertebral body can be restored for a bone amount of about 15% of the vertebral body which corresponds to a cement volume about 4 cc. The author showed that beyond 15% there is no significant increase in the stiffness of the vertebral body as well as in the pressure within the intervertebral disc. In addition, when the cement volume exceeds 15%, asymmetric distribution of the injected cement and excessive rigidity of the vertebral body can be caused.

Nieuwenhuijse et al. (2012), investigated the role of the injected cement dose in 196 patients with osteoporotic vertebral compression fracture by CT analysis after VP and showed that the average postoperative cement amount was about 3.94 mL for pain relief. Similar result were found by Belkoff et al. (2001) in his work indicating that restoration of the stiffness of the vertebral body in the thoracic and thoracolumbar regions required 4 mL. In their work, Kim et al., 2012 showed that bone cement restores vertebral stiffness when the cement volume reaches about 30% of the vertebral body for VP and that for higher volumes high increase of stiffness was observed which may increase spinal stresses.

The injection of the cement in combination with the implants generated a change of the vertebral body stiffness that affected the stress distribution within the spine segment (Figures 7–9, 11, 12). We can notice that V-STRUT technique generated a lower stress distribution compared to the VP one.

Our results showed a significant increase of the stress on the trabecular bone modulated with the cement dose (Figure 9) compared to the cortical one (Figure 8). We found that the stresses on the cortical/trabecular bone of the spine segment decreased for an injected cement dose in the range of 2-4 cc and increased in the range of 4-6 cc. Moreover, for a 4 cc cement volume, the stress contour is reduced suggesting that 4 cc is the optimal amount of bone cement to be injected. This is consistent with the 3D FE study of Wang et al. (2020) who found that 4 cc correspond to the optimal amount of injected to restore the injured spine during VP. Luo et al. (2009) confirmed that 3.5 cc of bone cement could restore the normal stress distribution of the vertebral body.

The general trend for all models is that stress in the augmented cortical bone do not change significantly because cement stiff the trabecular bone and hence, the stress is transferred to the cement and implants. Our results (Figure 9) indicated that for both VP and V-STRUT techniques, the stress level in the vertebral body located above the augmented one showed a greater increase compared to the vertebral body beneath with a stress level higher for VP when compared to the V-STRUT. This results conforms to the clinical study of (Trout and Kallmes, 2006; Boger et al., 2007). The authors showed that the vertebral body above the augmented one is more susceptible to undergoes fracture than the vertebra below.

This can be explained by the fact that the stiffnesses of the injected material (cement for VP and cement-implant assembly for V-STRUT) are greater of the cancellous bone stiffness and hence, tends to increase the stiffness of the treated augmented vertebra and the whole spine segment. Consequently, the stress in cancellous bone of the adjacent vertebras increases because the stiffening of the augmented vertebra reduced its cushioning capacity and hence, the adjacent vertebra mainly the above one are more stressed. Similar results was reported by Cho et al. (2015) indicating that increasing the stiffness of the treated vertebra increases the risk of adjacent vertebral fractures after VP in an osteoporotic FE model.

Figure 10 demonstrates that the V-STRUT solution transferred uniformly a part of the load to the posterior column. One can notice that for the current spine model the cement is subjected to a symmetric uniform stress distribution stabilized by the presence of the implants and that the VP generated a non-symmetric distribution. Liebschner et al. (2001) suggested that symmetric placement of the cement is an optimal configuration for the biomechanical response of the spine segment. Hence, one can expect that symmetric injected bone cement can lower the risk of adjacent fracture after injection. Chen et al. (2011) reported that the unilateral bone cement distribution generates a different stiffness to both sides of the vertebral, resulting in unbalanced stress. Contrary to the VP technique, V-STRUT device ensure bilateral injection of bone cement and hence, achieve a uniform distribution on both sides of the vertebral body, avoiding stress distribution difference which may cause by asymmetric distribution on the coronal plane (Cornelis et al., 2021; Barral et al., 2024).

The computed stress contour applied to the spine (Figure 11) and at the bone/implant interface clearly indicated that V-STRUT technique generated a uniform and lower stress distribution transferred to the trabecular bone compared to VP technique. This finding can have clinical implications in order to stabilize the fracture and reduce or suppress the pain.

Figure 12 shows that when the amount of the injected cement increases, the applied stress in the endplates of the augmented vertebra increases with a lower stresses values for V-STRUT technique compared to VP. The results indicates that the optimal cement volume ensuring reduced stress on the upper and lower endplates correspond to a value of 4 cc when using the V-STRUT device. For VP, 2 cc of cement generated a lower stress value at the upper endplates and 4 cc of cement for the lower endplates.

These results can have direct clinical implications when dealing with the optimal dose of the injected cement. It is also possible to select a particular volume value in order to assign a given (target) stiffness for a specific-patient. This contributes toward the personalized treatment. Further clinical studies are necessary to investigate the performance of the V-STRUT implant position to restore spine behavior.

Despite the rational construction of the current 3D FE models for VP and V-STRUT techniques, there are certain limitations. First, the sample size is limited to one osteoporotic spine segment, which yields large variation in the results and may not reflect the anatomical variability of the population. Differences in degree of osteoporosis in different patients may also result in variability. Nevertheless, the qualitative validity of the 3D FE model was obtained previously (Hambli et al., 2023), confirming the capability of the numerical model to predict representative responses. Current results indicated that 4 cc of bone volume is the optimal dose for the selected spine model. For clinical applications, patient-specific FE simulations are needed in order to assess the personalized dose of a given patient. A given (target) stiffness for a specific-patient. Second, our work implemented bilateral ellipsoid geometries of inserted cement which is not necessarily specific to patient-specific vertebral body or real-life fracture morphologies. Typically, different morphologies of cement were proposed by different authors. Nevertheless, ellipsoid capsules shapes were observed based on X-rays of different patients treated with the V-STRUT device. The third limitation concerns the loading mode. In the current study, the applied load was limited to the compressive physiological motions of the spine. Other modes of injury may need to be investigated. However, the V-STRUT device was designed principally to treat osteoporotic VCFs which are more often seen in “normal” activity and loading scenarios. Fourth, further studies are necessary to investigate the response of the V-STRUT^©^ implant under different loading cases. Fifth, the modelling of the discs behavior is based on macroscopic biomechanical model rather than mechanobiological one considering the etiology of intervertebral disc degeneration (IVD) as reported in the review study of (Volz et al., 2022). Nevertheless, several previously published 3D FE element models of the spine retained such a macroscopic description sufficient for general conclusions.

Considering enhanced models to describe the bone and discs behavior from a mechanobiological perspective is a necessary step towards the development of enhanced predictive simulations considering different factors such pathologies, aging and discs degenerations.

## 5 Conclusion

The 3D FE study showed that the bone cement volume has substantial effect on the biomechanical responses of treated osteoporotic spine segment with both VSTRUT and VP techniques. In the current FE investigation, it was found that the stresses on the trabecular bone of the spine segment decreased for an injected cement dose in the range of 2-4 cc and increased in the range of 4-6 cc. Based on these data, the VSTRUT device with a bilateral injected cement total of 4 cc is considered optimal to restore the stress and the stiffness of the augmented vertebra and related spine segment without excessively increasing the stresses on adjacent vertebrae in an osteoporotic spine.

The results showed the VSTRUT technique is superior to the VP one in terms of restoring the biomechanical behavior of an osteoporotic spine segment and reducing the transferred stress to the adjacent vertebra.

## Data Availability

The original contributions presented in the study are included in the article/supplementary material, further inquiries can be directed to the corresponding author.
